# Altered White Matter Integrity in Patients with Retinal Vein Occlusion: A Diffusion Tensor Imaging and Tract-Based Spatial Statistics Study

**DOI:** 10.1155/2022/9647706

**Published:** 2022-02-24

**Authors:** Mou-Xin Zhang, Min-Jie Chen, Li-Ying Tang, Chen-Yu Yu, Yu-Ling Xu, San-Hua Xu, Ping Ying, Min Kang, Li-Juan Zhang, Yi Shao

**Affiliations:** ^1^Department of Ophthalmology, The First Affiliated Hospital of Nanchang University, Jiangxi Province Ocular Disease Clinical Research Center, Nanchang, China; ^2^Affiliated Xiamen Eye Center of Xiamen University, School of Medicine, Xiamen University, Xiamen, China; ^3^Department of Clinical Medicine, Queen Mary School, Nanchang University, Nanchang, China; ^4^Department of Ophthalmology, Zhongshan Hospital of Xiamen University, School of Medicine, Xiamen University, Xiamen, China

## Abstract

**Background:**

To investigate microstructural alterations of white matter in retinal vein occlusion (RVO) patients by tract-based spatial statistics (TBSS) and diffusion tensor imaging (DTI). *Material/Methods*. DTI was performed on 14 RVO patients and 14 normal controls (HCs). We measured and recorded fractional anisotropy (FA) and radial diffusivity (RD) of white matter fibers and classified them through the receiver operating characteristic (ROC) curve and correlation analysis, respectively.

**Results:**

The mean FA value of white matter in RVO patients is lower than the HCs, and the mean RD value in RVO patients increased, especially in the bilateral posterior thalamic, bilateral sagittal stratum, body of corpus callosum, cingulum, and fornix. The ROC curve of different brain regions showed high accuracy. Moreover, the mean FA and RD values were significantly correlated with visual and psychological disorders.

**Conclusion:**

TBSS could be regarded as an important method to reveal the alterations of white matter in RVO patients, indicating the underlying neurological mechanism of the RVO.

## 1. Background

The retinal vein occlusion (RVO) is a kind of second major retinal vascular disorder, which is characterized by the expansion and dilation of retinal veins [1]. Meanwhile, RVO is considered to be an important cause of visual loss, including central (CRVO) and branch retinal vein occlusions (BRVO) [2, 3]. According to the previous epidemiological studies of world population, about 16 million adults have RVO, and the estimated prevalence rate of RVO is 5.2% [4]. RVO is closely related to the advancing age, which leads to its worldwide increase occurrence because of the increasing longevity of people [5]. RVO generally occurs in the case of thrombosis in the vascular system or some arterial diseases, which leads to intraluminal stenosis, venous congestion, and increased venous pressure [6]. This intravascular alteration potentially causes some secondary conditions including macular edema and neovascularization, which are the leading causes of vision loss in RVO [4, 6, 7]. Until now, there is still no complete and effective cure for RVO. A DTI study based on white matter suggests that retinal vascular pathology is related to poorer microstructure of cerebral white matter [8]. Thus, we speculate that some functional and microstructural changes may happen in the brain of patient with RVO. Currently, fundus fluorescein angiography (FFA) [1] and optical coherence tomography (OCT) [9] are often used for imaging diagnosis of the retinal and choroidal vasculature, but the neuroimaging examination is still rare. We believe that neuroimaging method may provide a new direction for revealing RVO-related brain processes and the potential neuropathological mechanisms.

DTI is a special form of functional magnetic resonance imaging (MRI) and is a new way to describe the structure of the brain in recent years. As a new imaging method based on diffusion weighted imaging (DWI), DTI can analyze the dispersion motion of water molecules in the tissue in three-dimensional space, providing an insight into living human brain noninvasively, especially white matter anatomy. Additionally, it gives quantitative parameters related to the microstructure of white matter [10, 11]. A number of measures calculated using DTI measures can provide quantitative information, including FA, RD, axial diffusivity (AD), and mean diffusivity (MD) [12, 13]. Many studies successfully used DTI to demonstrate intrinsic neural alterations in patients with visual-related diseases. Li et al. [14] found that FA of optic nerve and optic nerve radiation decreased significantly, while MD of bundle increased significantly, suggesting that indicative degeneration and remodeling may occur in patients with glaucoma. Besides, Li et al. [15] applied voxel-wise statistical analysis and reported that there is a significant decrease FAs in several white matter tracts and reduced grey matter volume in children with amblyopia compare to HCs, which revealed that visual impairment of amblyopia affects normal development of brain structure. However, the intrinsic changes of white matter in RVO patients remain unclear. RVO could cause vision loss, which in turn may lead to mental illness such as anxiety and depression. Thus, we suspected that the average FA and RD might be related to anxiety, depression, and vision.

Because of the advantages of voxel and trajectory analysis, trajectory-based spatial statistics (TBSS) is not only widely used in various neurological system diseases but also has become a popular tool to evaluate DTI data. TBSS projects volumetric data onto a WM skeleton without data smoothing, resolving the defect caused by alignment inaccuracies [16, 17]. Previous studies have applied TBSS to assess the neural alterations in patients with visual-related diseases. A TBSS analysis of patients with concomitant exotropia showed that FA increased and RD decreased in the relevant areas [18]. TBSS was applied to investigate the white matter integrity of patients with congenital and terminal blind and monocular blindness and found the different mechanisms of structural changes in white matter [19, 20]. Therefore, our purpose here is to use TBSS analysis to research the alterations and to explore the diffusivities of fiber bundles in RVO patients.

## 2. Methods

### 2.1. Subjects

**Figure 1:**
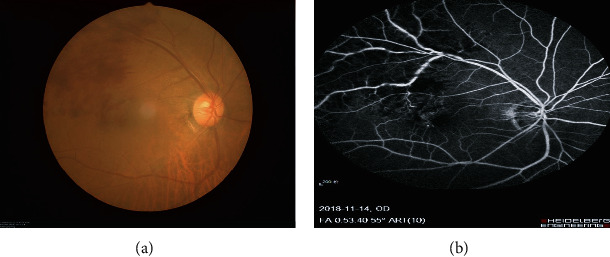
Example of RVO seen on FC and FFA. (a) RVO observed using a FC which was characterized by massive flame-like hemorrhage (red arrow) in the retina. (b) RVO seen on FFA which was characterized by petal-shaped fluorescein leakage (red arrow) in the retina. RV: retinal vein occlusion. FC: fundus camera. FFA: fluorescence fundus angiography.

We recruited 14 RVO patients from the First Affiliated Hospital of Nanchang University Hospital. The inclusion criteria for this study included the following: [1] ophthalmoscopy showed RVO signs; [2] OCT revealed macular oedema; and [3] FFA showed occlusion of retinal vein (Figure 1 and Table 1). The exclusion criteria for RVO were as follows: [1] a history of intraocular or extraocular surgery; [2] combined with other ocular diseases; and [3] mental disease, cardiovascular diseases, and other systematic diseases.

At the same time, 14 healthy controls (HCs) were included with the following inclusion criteria: [1] no history of ocular disease; [2] no brain abnormalities; [3] no mental and cardiovascular diseases; [4] no drugs or alcohol abuse; and [5] have MRI examination ability. The age and gender background of all HCs and RVO were matched. This study was conducted in strict accordance with the guidelines and regulations of the Human Research Ethics Committee of the First Affiliated Hospital of Nanchang University on the basis of approval. All patients included in this study were informed and signed a consent form.

### 2.2. Data Acquisition and Preprocessing

The data in this study was collected by 3.0 T MRI scanner (Siemens, Erlangen, Germany). The parameters of these sequences are referenced in previous studies [18]. FMRIB Software Library (FSL) was used for all MRI data. We performed these data as previously described [18]. All original data were extracted, corrected eddy current distortion and head motion artifacts, and then a brain mask was made.

### 2.3. TBSS Procedures

Similar to the previous TBSS method [16], we used the following analytical methods to explore the characteristics of white matter diffusion. All FA images were aligned with the standard space of a Montreal Neurological Institute 152 (MNI152) through nonlinear registration. Firstly, the mean FA maps of all participants were projected onto the fMRIB 58 skeleton. Then, after maximum alignment of the common skeleton, the data was presented as a four-dimensional image. We used the FSL view and FSL cluster tool to visualize and select the statistically significant FA and RD voxel clusters, respectively, and simplified the visualization of the actual analytical representation through the script TBSS_fill.

### 2.4. Evaluation of Anxiety, Depression, and Visual quality

The Hospital Anxiety and Depression Scale (HADS) designed in 1983 was used to investigate anxiety and depression [21]. The Chinese version of National Eye Institute 25-Item Visual Function Questionnaire (NEI-VFQ25) was used to measure the quality score of life [22].

### 2.5. Receiver Operating Characteristic Curve (ROC)

We analyzed the average values of FA and RD through the ROC methods as previously described [20]. The area under the curve (AUC) represented the diagnostic rate. The AUC value of 0.7~0.9 and 0.5~0.7 represented lower and higher accuracy, respectively.

### 2.6. Statistical Analysis

The clinical and demographic variables were analyzed by two-independent-sample *t*-tests of SPSS 23.0 (IBM Corp., USA). Threshold-free cluster enhancement option in the FSL randomize tool was used to synchronously implement the nonparametric method based on permutation. *P* < 0.05 indicated that the results were statistically significant through multiple complete corrected comparisons.

## 3. Results

The demographic and clinicopathological factors of patients are shown in Table 1. The average ages of RVO patients and HCs were 52.22 ± 5.95 and 52.12 ± 5.01, respectively. The average weights of RVO patients and HCs were 51.23 ± 9.11 and 52.35 ± 10.09, respectively.

**Figure 2:**
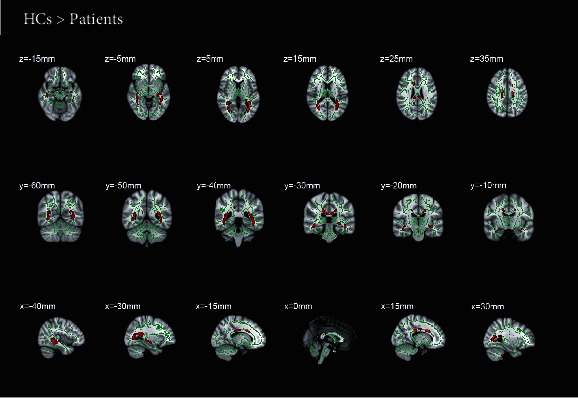
Results of whole-brain tract-based spatial statistics analysis comparing fractional anisotropy between RVO patients and HCss. The skeleton image (green = RD > 0.2) was overlaid by the mean fractional anisotropy image. And the red areas indicate all tracts with significantly decreased RD values in the RVO patients, which may reflect abnormal white matter integrity (*p* < 0.05). Significantly lower fractional anisotropy values were shown in the body of corpus callosum, right posterior thalamic radiation, left posterior thalamic radiation, right sagittal stratum, left sagittal stratum, right cingulum, left fornix/stria terminalis. RVO: retinal vein occlusion. HCs: healthy controls.

**Figure 3:**
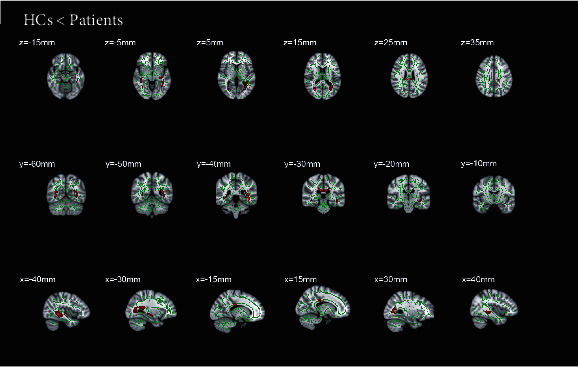
Comparison of radial diffusivity in RVO patients and HCs. The skeleton image (green = RD > 0.2) was overlaid by the mean fractional anisotropy image. And the red areas indicate all tracts with significantly increased RD values in the RVO patients, which may reflect abnormal white matter integrity (*p* < 0.05). The statistically significant clusters are presented at different coordinates in these six parts. These clusters include body of corpus callosum, right posterior thalamic radiation, left posterior thalamic radiation, right sagittal stratum, left sagittal stratum, right cingulum, left fornix/stria terminalis. RVO: retinal vein occlusion. HCs: healthy controls.

The comparison of voxel clusters between two groups in FA and RD presented a notably difference. The declining mean FA value of total cerebrum in RVO group was detected (Table 2). Additionally, the mean RD value of total cerebrum in RVO patients was higher than that in HCs (Table 3). The patients showed significantly lower FA values and higher RD values in five clusters: the bilateral posterior thalamic radiation, bilateral sagittal stratum, body of corpus callosum, cingulum, and fornix (Figures 2 and 3).

### 3.1. ROC Curve

**Figure 4:**
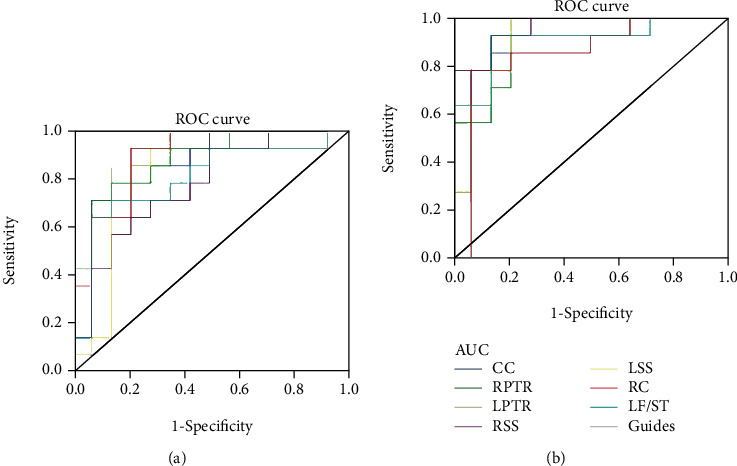
ROC curve analysis of the mean FA and RD values for altered brain regions. (a) The areas under the ROC curve for the FA values were as follows: the body of corpus callosum (CC), 0.862 (*p* < 0.001; 95% CI: 0.719–1.000); the right posterior thalamic radiation (RPTR), 0.883 (*p* < 0.001; 95% CI: 0.759–1.000); the left posterior thalamic radiation (LPTR), 0.959 (*p* < 0.001; 95% CI: 0.896–1.000); the right sagittal stratum (RSS), 0.770 (*p* < 0.001; 95% CI: 0.595–0.946); the left sagittal stratum (LSS), 0.837 (*p* < 0.001; 95% CI: 0.667–1.000); the right cingulum (RC), 0.893 (p < 0.001; 95% CI: 0.773–1.000); the left fornix/stria terminalis (LF/ST), 0.796 (*p* < 0.001; 95% CI: 0.620–0.971). (b) The areas under the ROC curve for the RD values were as follows: the body of CC, 0.923 (*p* < 0.001; 95% CI: 0.816–1.000); the RPTR, 0.918 (*p* < 0.001; 95% CI: 0.817–1.000); the LPTR, 0.969 (*p* < 0.001; 95% CI: 0.917–1.000); the RSS, 0.959 (*p* < 0.001; 95% CI: 0.896–1.000); the LSS, 0.908 (*p* < 0.001; 95% CI: 0.792–1.000); the RC, 0.847 (*p* < 0.001; 95% CI: 0.685–1.000); the LF/ST, 0.908 (*p* < 0.001; 95% CI: 0.792–1.000). AUC: area under the curve, FA: fractional anisotropy, RD: radial diffusivity, ROC: receiver operating characteristic, CC: corpus callosum, RPTR: right posterior thalamic radiation, LPTR: left posterior thalamic radiation, RSS: right sagittal stratum, LSS: left sagittal stratum, RC: right cingulum, LF/ST: left fornix/stria terminalis.

The areas under the ROC curve for the FA values were as follows: the body of corpus callosum (CC), 0.862 (*p* < 0.001; 95% CI: 0.719–1.000); the right posterior thalamic radiation (RPTR), 0.883 (*p* < 0.001; 95% CI: 0.759–1.000); the left posterior thalamic radiation (LPTR), 0.959 (*p* < 0.001; 95% CI: 0.896–1.000); the right sagittal stratum (RSS), 0.770 (*p* < 0.001; 95% CI: 0.595–0.946); the left sagittal stratum (LSS), 0.837 (*p* < 0.001; 95% CI: 0.667–1.000); the right cingulum (RC), 0.893 (*p* < 0.001; 95% CI: 0.773–1.000); and the left fornix/stria terminalis (LF/ST), 0.796 (*p* < 0.001; 95% CI: 0.620–0.971) (Figure 4(a)).

The areas under the ROC curve for the RD values were as follows: the body of CC, 0.923 (*p* < 0.001; 95% CI: 0.816–1.000); the RPTR, 0.918 (*p* < 0.001; 95% CI: 0.817–1.000); the LPTR, 0.969 (*p* < 0.001; 95% CI: 0.917–1.000); the RSS, 0.959 (*p* < 0.001; 95% CI: 0.896–1.000); the LSS, 0.908 (*p* < 0.001; 95% CI: 0.792–1.000); the RC, 0.847 (*p* < 0.001; 95% CI: 0.685–1.000); the LF/ST, 0.908 (*p* < 0.001; 95% CI: 0.792–1.000) (Figure 4(b)).

### 3.2. Correlation Analysis

**Figure 5:**
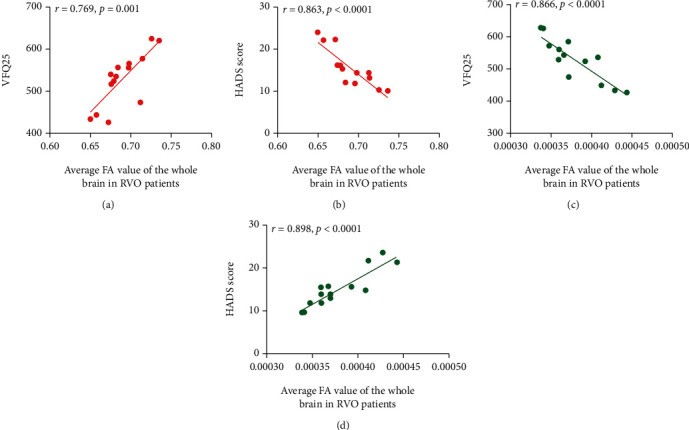
Correlation between the average FA and RD values of the whole brain in RVO patients and anxiety, depression and NEI-VFQ25 scores. (a) The average FA value of the whole brain was positively correlated with the NEI-VFQ25 score (*r* = 0.769, *p* = 0.001). (b) The average FA value of the whole brain was negatively correlations with the HADS score (*r* = −0.863, *p* < 0.0001). (c) The average RD value of the whole brain displayed negatively correlated with the NEI-VFQ25 score (*r* = −0.866, *p* < 0.0001). (d) The average RD value of the whole brain displayed positively correlations with the HADS score (*r* = 0.898, *p* < 0.0001). RVO: retinal vein occlusion. FA: fractional anisotropy. RD: radial diffusivity.

The average FA value of the whole brain was positively correlated with the NEI-VFQ25 score (*r* = 0.769, *p* = 0.001) and negatively correlated with the HADS score (*r* = −0.863, *p* < 0.0001) in RVO patients. The mean RD value of the total cerebrum negatively correlated with the NEI-VFQ25 score (*r* = −0.866, *p* < 0.0001) and positively correlated with the HADS score (*r* = 0.898, *p* < 0.0001) (Figure 5).

## 4. Discussion

This study is the first TBSS analysis of RVO patients. We adopted DTI scanning and TBSS analysis to probe the fiber bundle architecture differences between the objective group and the HCs. To be brief, our findings were that there were significant differences in voxel clusters, and RVO patients showed lower FA values than the other group, whereas higher RD values were presented.

Until now, the TBSS approach for DTI analysis has been widely used in neuroimaging studies. Compared to the statistical parametric mapping–based approach, TBSS averts the insufficiency about registration and smoothing of diffusion data. Furthermore, in TBSS, we can investigate the brain globally rather than specified certain tracts [16, 23].

In this study, DTI and TBSS were applied in RVO patients and we found reduced FA and increased RD in the B.PTR (involve the optic radiation) which is relevant for the connection between the thalamus and the visual cortex [24]. Previous voxel-based morphometry showed that reduced gray matter density in the occipital lobe has been demonstrated in patients with diabetic retinopathy [25]. Malania et al. [26] have found significant diffusion abnormalities along the visual pathways in patients with macular degeneration. Additionally, low FA in optic tract was especially detected in patients with retinitis pigmentosa in a previous study [27]. As the transmission of visual information involves the whole visual system. It is assumed that the damage at retina could trigger microstructural changes in brain. FA value is influenced by various neurostructural factors and a decrease in FA was interpreted as structural damage in nerve bundle [28]. Consistent with the above results, the decreased FA in our study indicated that the integrity of tracts was impaired in RVO patients. An increase in RD indicated the destruction of cell structure and myelin damage [29, 30]. We further infer that an increase in RD suggests that some pathologic alterations in PTR, representing the underlying neuropathologic mechanism. Consequently, we further deduce that this alteration might lead to visual damage in RVO patients.

The sagittal stratum, a large sagittal structure, consists of inferior longitudinal fasciculus (ILF), inferior fronto-occipital fasciculus (IFO), and other projection fibers [24, 31]. ILF and IFO are closely related to visual information processing. The ILF provides pivotal connections between occipital and anterior temporal regions and also is closely associated with the optic radiations. A study focusing on functional anatomy of the ILF suggested that it mediated process of visual memory and recognition of visual information [32, 33]. The IFO was considered to connect with different cortical regions within the frontal, occipital, and temporal lobes. Study have once showed that IFO have a critical meaning in visual processing and reading [34, 35]. In similar visual neuroscience studies, Li et al. [15] used DTI method and indicated significant decreases in FA values in left ILF/IFO in children with anisometropic amblyopia. Li D. et al. [18] found an increased FA values in ILF and IFO in patients with comitant exotropia. Furthermore, Cheng et al. [36] reported that dyslexia may related to visual perception deficits. In current study, changed FA and RD values of SS suggested that relevant nerve tract was damaged in RVO patients, indicating visual information processing disorder and visual dysfunction. On the other hand, it was affirmed that the higher order visual processing systems of normal individuals is hierarchically organized into two functionally systems, the dorsal and ventral visual pathway [37]. The ventral stream consists of the IFO and the ILF principally, which is relevant to object recognition [38]. A DTI study has shown that the microstructure of the ventral stream changed in blind patients [39]. Therefore, the alterations in FA and RD values of sagittal stratum might clarify the visual impairment in RVO patients.

The corpus callosum is located at the base of the longitudinal fissure of the cerebral hemisphere. As the largest bundle of connective fibers in the human brain, it connects cortical regions of both hemispheres [40]. The major function of CC is to integrate information such as motor sensation and cognitive activity between two cerebral hemispheres [41]. Reports in the literature indicated that the CC has an intimate connection to the visual cortex. The well connectivity of the callosal fibers is influenced by alterations of visual input [42]. Kwinta et al. detected low FA values of the CC in premature infants with abnormal stereoscopic vision and visual perception [43]. Consistent with previous researches, we speculated that the decrease of FA and increase of RD indicated the integrity of inner structure of the CC was damaged and potential pathologic changes or degeneration may occur in the callosal fibers in RVO patients, which may leads to inability to integrate visual information and further cause visual deprivation.

Interestingly, in addition to vision-related regions, we also found changed FA and RD values in extravisual-related regions including cingulum and fornix. As one of the most distinctive fiber tracts in the brain, cingulum connects frontal, parietal, and temporal sites. The largest efferent pathway in the hippocampus is the fornix, which is the critical component of the limbic system of brain [44, 45]. According to previous studies, significantly decreased FA value of cingulum was discovered in depression patients [44]. McCarthy-Jones et al. [46] found reduced integrity of fornix in the process of childhood adversity. Therefore, the limbic system plays an important role in regulating sensory information in the central nervous system. Previous studies have confirmed that patients with visual field and acuity loss have psychological and emotional disorders [47, 48]. On the basis of the above studies, the decrease of FA value in RVO patients may reflect the injury of nerve tracts related to emotion management. Moreover, Schafer et al. [48] have discovered that the autistic neurons grow faster and have more complex branches. We speculate that the increased RD values in correlative tracts may clarify the neuropathologic mechanism of RVO, which tries to repair nerve tract damage by enhancing neuronal branching to improve their function. Hence, visual impairment defects increase the psychological disorders of RVO patients. Our findings may contribute to illustrate the causes of negative emotions in RVO patients. In addition to treatment at the disease itself, focusing more on the psychotherapy may improve the prognosis of RVO patients.

Our current research has some boundedness. First, the sample capacity of RVO subjects is relatively small. We need larger samples for further study. Secondly, there are many aetiological causes of RVO, which may cause individual differences and lead to imprecise results of TBSS measurements. Additionally, we merely analyze the diffusivities separately and we believe it is necessary to investigate the interaction between FA and RD values.

## 5. Conclusion

It was shown that some structural alterations of correlative tracts developed, which may reveal the neuropathologic mechanism and may be responsible for the visual impairment in RVO patients. Overall, DTI with the TBSS method is an available tool to indicate the potential intracephalic tract involvement in RVO patients.

## Figures and Tables

**Table 1:** Demographics and clinical measurements of RVO and HC groups.

Characteristic	RVO	HC
Male/female	8/6	8/6
Age (years)	52.22 ± 5.95	52.12 ± 5.01
Weight (kg)	51.23 ± 9.11	52.35 ± 10.09
Handedness	14R	14R

RVO: retinal vein occlusion. HC: healthy control. N/A: not applicable. R: right. L: left.

**Table 2:** Clusters showing significant differences in FA between RVO patients and HCs.

Variable	Comparison	TFCE corrected *p*	Cluster number	MNI coordinates			*T* values
FA	RVO<HCs	<0.01		*X*	*Y*	*Z*	
	BCC		1	81	96	96	−6.254
	RPTR		2	58	83	89	−5.185
	LPTR		3	119	62	90	−6.025
	RSS		4	53	71	67	−4.187
	LSS		5	130	89	61	−5.029
	RC		6	79	79	97	−5.162
	LF		7	116	96	68	−4.587

RVO: retinal vein occlusion. HC: healthy control. BCC: body of corpus callosum. RPCR: right posterior corona radiata. LPTR: left posterior thalamic radiation. RSS: right sagittal stratum. LSS: left sagittal stratum. RC: right cingulum. LF: left fornix.

**Table 3:** Clusters showing significant differences in RD between RVO patients and HCs.

Variable	Comparison	TFCE corrected *p*	Cluster number	MNI coordinates			*T* values
	Patients > HCs			*X*	*Y*	*Z*	
RD	BCC	<0.01	1	80	96	96	5.256
	RPTR		2	58	65	72	4.298
	LPTR		3	117	63	87	6.248
	RSS		4	51	91	68	3.284
	LSS		5	130	89	61	4.096
	RC		6	82	96	103	5.267
	LF		7	121	100	66	5.019

RVO: retinal vein occlusion. HC: healthy control. BCC: body of corpus callosum. RPCR: right posterior corona radiata. LPTR: left posterior thalamic radiation. RSS: right sagittal stratum. LSS: left sagittal stratum. RC: right cingulum. LF: left fornix.

## Data Availability

The data used to support the findings of this study are available from the corresponding author upon request.

## Abbreviations

RVO: Retinal vein occlusion
FC: Fundus camera
FFA: Fluorescence fundus angiography
HCs: Healthy controls
AUC: Area under the curve
FA: Fractional anisotropy
RD: Radial diffusivity
ROC: Receiver operating characteristic
CC: Corpus callosum
RPTR: Right posterior thalamic radiation
LPTR: Left posterior thalamic radiation
RSS: Right sagittal stratum
LSS: Left sagittal stratum
RC: Right cingulum
LF/ST: Left fornix/stria terminalis.
